# 

**Published:** 1895-10

**Authors:** 


### CONTENTS.

ORIGINAL COMMUNICATIONS—

THE MEDICAL ASPECT OF TREPHINING IN EPILEPSY. By Eugene Foster, M.D., Augusta, Ga. .... 449

RICKETS. By Charles S. Shaw, M.D., Pittsburg, Pa. .... 466

THERAPEUTICS OF HEART DISEASE. By Horatio C. Wood, M.D., LL.D., Philadelphia, Pa .... 472

ECLAMPSIA AFTER LABOR. By D. A. York, M.D., Cornelia, Ga. .... 481

CORRESPONDENCE—

NEW YORK LETTER .... 482

RADICAL TREATMENT OF DISEASES OF NOSE AND THROAT .... 487

EDITORIAL—

DR. ROBERT BATTEY .... 489

FAKIR JOURNALISM .... 490

MEDICINE AS SHE IS LEARNED .... 491

TO SUBSCRIBERS—IMPORTANT .... 492

SELECTIONS AND ABSTRACTS—

SURGERY .... 493

SKIN DISEASES AND SYPHILIS .... 497

GENERAL MEDICINE AND THERAPEUTICS .... 501

MEDICAL ITEMS .... 507

BOOK REVIEWS .... 509

ADVERTISERS' INDEX TO ADVERTISEMENTS .... xiv

MEDICAL ITEMS—CONTINUED .... x, xi, xii, xiii

CLASSIFIED INDEX TO ADVERTISEMENTS .... xxxv

